# Comment on Moriconi et al. Very-Low-Calorie Ketogenic Diet as a Safe and Valuable Tool for Long-Term Glycemic Management in Patients with Obesity and Type 2 Diabetes. *Nutrients* 2021, *13*, 758

**DOI:** 10.3390/nu13103613

**Published:** 2021-10-15

**Authors:** Kirsten A. Berk, Elles J. T. M. van der Louw, Joanne F. Olieman, Aart J. van der Lely

**Affiliations:** Department of Internal Medicine, Erasmus University Medical Center, 3015 GD Rotterdam, The Netherlands; e.vanderlouw@erasmusmc.nl (E.J.T.M.v.d.L.); j.olieman@erasmusmc.nl (J.F.O.); a.vanderlelij@erasmusmc.nl (A.J.v.d.L.)

With interest, we have read the article of Moriconi et al. [1] entitled “Very-Low-Calorie Ketogenic Diet as a Safe and Valuable Tool for Long-Term Glycemic Management in Patients with Obesity and Type 2 Diabetes”. A very low carbohydrate ketogenic diet (VLCHKD; <10% energy from CHO/day) is a popular, often effective dietary intervention for people with obesity related-type 2 diabetes mellitus (T2DM) [2,3,4,5,6]. However, ketogenic diets applied in T2DM studies differ significantly in nutrient composition (different percentages of carbohydrate, protein, fat and types of fatty acids), resulting in a heterogeneity that influences reproducibility and interpretation of their results.

Moriconi et al. [1], did not provide data on the nutritional composition of the VLCHKD during the study period, making the interpretation and translation of the results into everyday practice difficult. Furthermore, the title of the paper indicates that ketosis was reached, but data on the achieved ketosis are not presented. As studies among adults with diabetes often opt for a diet with a ratio of fat to carbohydrates and protein of no more than 1:1, the question is whether substantial ketosis actually occurred during the intervention in the study of Moriconi and co-workers. This is in contrast to diets used, e.g., in children with epilepsy, in which ratios of 3:1 and higher are often achieved, resulting in a considerable level of ketosis [7,8].

In addition, we have some concerns about the methodology used in the study by Moriconi et al. [1]. First of all, the significant differences in baseline anthropometric measures between the cohorts certainly influence the results. For example, the VLCHKD group had 20 kg more excess in bodyweight and thus presumably this cohort had a higher motivation and potential for improvement. It is also unclear whether participants were free to choose the dietary intervention. Moreover, details on the time period in which the data from the historical cohort were collected are missing.

The use of VLCHKDs is an important and promising area of research in obesity-related T2DM. However, interpretation of the available published results is currently hampered by differences in diet composition, incomplete reporting of diet composition, and differences in nomenclature as also indicated by Trimboli et al. [9]. We therefore strongly advocate a consensus on these issues in the field of obesity-related T2DM in order to improve the quality of study designs, interpretation and reproducibility of data. Although the results reported by Moriconi et al. [1] are of interest, we believe that they should be interpreted with caution.
